# Sustainable Graphite and Jet Fuel from Biorefinery Residue

**DOI:** 10.1002/cssc.202402509

**Published:** 2025-03-12

**Authors:** Lillian Lower, Steven M. Rowland, Michael Regula, Kristiina Iisa, Zachary A. Combs, Sunkyu Park, Tijmen Vries, Ton Vries, Mark R. Nimlos, William Joe Sagues

**Affiliations:** ^1^ Department of Biological and Agricultural Engineering North Carolina State University Raleigh, NC 27695 USA; ^2^ National Renewable Energy Laboratory Golden, CO 80401 USA; ^3^ Birla Carbon Marietta, GA 30062 USA; ^4^ Department of Forest Biomaterials North Carolina State University Raleigh, NC 27695 USA; ^5^ BioBTX Groningen Netherlands

**Keywords:** Li-ion battery, graphite anode, biomaterials, sustainable aviation fuel, pyrolysis

## Abstract

Battery‐grade graphite and aviation fuel are traditionally produced from non‐renewable, fossil carbon feedstocks and result in substantial greenhouse gas emissions. Biomass holds exciting potential as a renewable and sustainable feedstock for the production of graphite and aviation fuel, but challenges exist including the necessity of a catalyst when producing graphite and low selectivity when producing aviation fuel. A process to convert a biomass‐derived feedstock into graphite without the use of a catalyst and fuels with high selectivity towards sustainable aviation fuel (SAF) is innovated. Heavy bio‐oil undergoes a conversion process similar to the commercial production of synthetic graphite including coking at 500 °C, calcination at 1000 °C, and graphitization at 2800 °C. The resulting biographite exhibits excellent performance in lithium‐ion battery configurations with specific capacity of ~330 mAh g^−1^ and a 96.8 % capacity rebound after high rate cycling. The liquid hydrocarbon co‐product from coking is suitable for hydrotreating into SAF. The aviation fuel fraction (70 wt % of the fuel produced) meets ASTM standards and is composed primarily of cycloalkanes (~80 wt %) which improves energy density compared to paraffins produced by other SAF pathways and may replace aromatics for elastomer swelling in traditional jet fuel with less soot production.

## Graphical abstract

This study explores the novel conversion of bio‐oil from benzene‐toluene‐xylene (BTX) production into biographite and sustainable aviation fuel (SAF). By replicating synthetic graphite production through coking, calcination, and graphitization, the process yields high‐performance biographite suitable for lithium‐ion batteries. Additionally, the co‐produced fuel consists of 70 wt % SAF, rich in high‐energy‐density cycloalkanes, providing a cleaner alternative to conventional jet fuel.

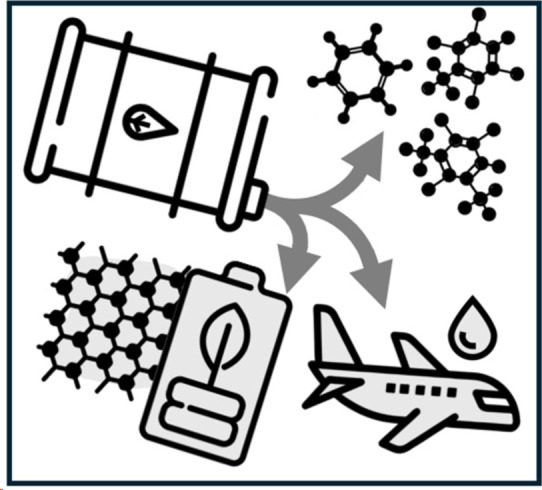

## Introduction

Catalytic fast pyrolysis is a promising thermochemical biorefining process that converts biomass‐derived feedstocks into high‐value chemicals, including the aromatic commodity chemicals benzene, toluene, and xylene (BTX). Unlike traditional fast pyrolysis, catalytic fast pyrolysis integrates the thermochemical decomposition of biomass with catalytic upgrading, resulting in bio‐oils with significantly reduced oxygen content, making them more suitable for hydrocarbon production, including BTX. One promising biomass‐derived feedstock for catalytic fast pyrolysis is crude glycerol, or glycerin, which constitutes approximately 10 wt % of total outputs from industrial biodiesel production.[^1^, ^2^, ^3^] Due to its unique composition and availability, glycerol has been identified as a feedstock for catalytic pyrolysis and demonstrates high BTX yields.[^4^, ^5^, ^6^, ^7^, ^8^] Specifically, Integrated Cascading Catalytic Pyrolysis (ICCP) has shown potential for BTX production from glycerol. ICCP employs a zeolite catalyst (e. g. H‐ZSM‐5) to upgrade pyrolysis vapors, producing an oil rich in aromatics with low oxygenate content.[^9^, ^10^, ^11^, ^12^, ^13^, ^14^] While the BTX can be separated from the oil by distillation, the non‐BTX residues are more difficult to use for high‐value products due to their elevated oxygen content and high concentration of polycyclic aromatic hydrocarbons (PAHs).[^1^, ^9^, ^10^, ^15^, ^16^, ^17^, ^18^, ^19^, ^20^, ^21^, ^22^, ^23^, ^24^] Efforts to enhance BTX production have focused on optimizing pyrolysis parameters, catalyst selection, and upgrading strategies. However, a challenge remains: the valorization of PAH‐rich residues. Previous research explored hydrotreating PAHs to produce monoaromatics or utilizing PAHs in aluminum‐ion battery cathode applications.[^25^, ^26^, ^27^] Notably, Genuino *et al*. (2019) conducted ICCP of crude glycerol, obtained BTX and PAH fractions, and hydrogenated the PAH fraction using a Ru/C catalyst. The hydrogenated PAH fraction was then added to the crude glycerol feedstock and recycled into the system, resulting in an increase in BTX yields by 17 wt %.^[9]^

**Figure 1:**
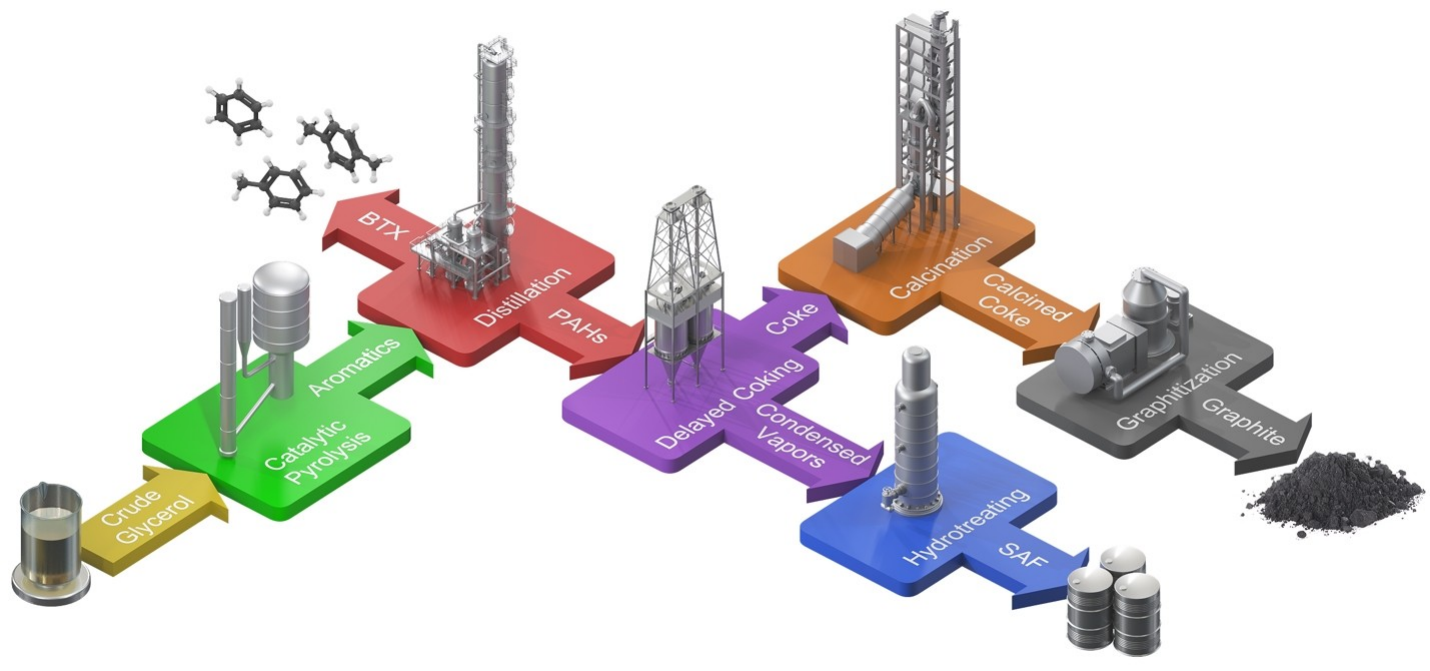
Simplified process flow diagram showing the production of sustainable aviation fuel (SAF) and biographite from bio‐oil containing polyaromatic hydrocarbons (PAHs), which is a by‐product from the catalytic pyrolysis of crude glycerol for benzene, toluene, and xylene (BTX) production.

In contrast to existing approaches, this study presents a novel valorization pathway for PAH‐rich residues by transforming them into battery‐grade graphite and sustainable aviation fuel (SAF) as illustrated in Figure 1. This approach leverages the heavy, highly aromatic PAHs that would typically be hydrotreated to low‐value products, repurposing them into biographite with comparable quality to commercial graphite for lithium‐ion battery anodes. We demonstrate that bio‐oil, derived from ICCP of glycerol can be processed into biographite via traditional, non‐catalytic conversion pathways, positioning ICCP‐bio‐oil and its resulting bio‐coke as a viable drop‐in replacement for petroleum‐derived needle coke in synthetic graphite production.

Graphite is the predominant active material in commercially‐available lithium‐ion battery anodes, commonly sourced as a mixture of natural, or mineral, and synthetic graphite, due to its availability and well‐characterized electrochemical properties.[^28^, ^29^] With the increasing demand for lithium‐ion batteries, the global graphite market is projected to expand from $24 billion to $42 billion by 2032^[30]^. Supply chain constraints on both natural and synthetic graphite necessitate alternative graphite precursors to meet anticipated demands.[^31^, ^32^, ^33^] Natural graphite is a nonrenewable resource that relies on scarce, geographically‐constrained deposits, costly extraction, and extensive environmentally detrimental purification requirements.[^29^, ^34^, ^35^, ^36^, ^37^] Conversely, synthetic graphite is produced from highly aromatic fossil carbons, such as petroleum‐derived fluid catalytic cracking (FCC) slurry oil, undergoing a three step thermal treatment: coking at 500 °C to produce green coke, calcination at 1500 °C to produce calcined, or needle, coke and graphitization at 2800 °C.[^29^, ^38^, ^39^] Relative to natural graphite, synthetic graphite is a more consistently‐crystalline material that is more expensive but increasingly becoming preferred by lithium‐ion battery manufacturers because of its small particle size and superior electrochemical performance.^[29]^ The growing need for accessible and renewable battery‐grade graphite materials has spurred innovative solutions, including the creation of sustainable biographite from widely available biomass materials.[^29^, ^40^]

The key challenge for direct biographite production is that most biomass precursors fail to undergo graphitization at high temperatures (~3000 °C), instead forming disordered hard carbon materials. As a result, biomass materials have been largely classified as “non‐graphitizing carbons”.[^41^, ^42^] A limited number of studies report graphitic carbon formation via high temperature, non‐catalytic treatment of biomass materials, but these materials exhibit mixed structures with lower crystallinity than commercial graphite. Additionally, few studies have evaluated these materials in lithium‐ion battery applications.[^41^, ^43^, ^44^, ^45^, ^46^, ^47^] To overcome this limitation, catalytic graphitization using transition metals (e. g., nickel, magnesium, iron) has been employed to facilitate the transformation of biomass into crystalline graphite at lower temperatures (~1500 °C).[^29^, ^40^, ^42^, ^48^, ^49^] However, this process, unlike traditional synthetic graphite production, requires the addition and subsequent removal of a catalyst, including acid leaching and microwave digestion, which hinder its scalability and commercial viability.[^29^, ^32^] Purified biographite materials generally require the same milling and shaping processes as natural and synthetic graphite used in lithium‐ion applications. However, emerging biographite synthesis methods may eliminate the need for traditional milling and shaping.^[50]^ The additional catalyst application and purification steps prompt manufacturing modifications to existing synthetic graphite production systems and have inhibited this process from surpassing a technology readiness level of 4.[^28^, ^51^] The need for a catalyst‐free route to battery‐grade biographite production that aligns with existing synthetic graphite manufacturing processes remains a critical knowledge gap that could significantly reduce the lithium‐ion battery industry's dependance on nonrenewable resources if filled. This study addresses this gap by demonstrating, for the first time, a three‐step heat treatment (coking, calcination, and graphitization) of a bio‐based feedstock that mirrors conventional synthetic graphite production without the need for catalysts. Additionally, we demonstrate that the condensable vapors produced during coking can be hydrotreated to yield a hydrocarbon fuel rich in sustainable aviation fuel (SAF), presenting a dual valorization strategy for ICCP‐derived PAH residues.

The feedstock used in this study originates from ICCP of biomass‐derived glycerol and consists of bio‐oil that is rich in BTX and PAHs. At low biomass‐to‐catalyst ratios, ICCP generates a bio‐crude abundant in monoaromatics (BTX) and PAHs; the latter considered a low‐value byproduct[^9^, ^52^]. Following BTX separation, the residual PAH‐rich oil closely resembles the precursor for petroleum‐derived needle coke, the primary feedstock for synthetic graphite.[^53^, ^54^] In petroleum refining, FCC distillation residuals that contain high levels of PAHs serve as the primary source for needle coke production.[^29^, ^39^] By leveraging PAH‐rich ICCP bio‐oil as an alternative feedstock, this work establishes a pathway for biographite production that integrates seamlessly into existing graphitization infrastructure, avoiding the extensive process modifications required by catalytic graphitization approaches.

Beyond biographite production, this novel valorization pathway yields a second high‐value product: SAF. Sustainable aviation fuel is essential for mitigating greenhouse gas emissions from the aviation sector, which is expected to continue expanding globally.[^55^, ^56^] During the coking of PAH‐rich bio‐oil, approximately 80 % of the initial sample mass is condensed as liquid that, upon hydrotreatment, produces a hydrocarbon fuel composed of ~70 % SAF. Notably, the SAF produced from this ICCP oil is composed primarily of cycloalkanes, a desirable fuel component due to their higher energy density compared to paraffins.^[57]^ Furthermore, unlike conventional paraffinic SAF, cycloalkane‐rich SAF retains the elastomer swelling properties of aromatic compounds in traditional jet fuel, without generating excessive soot upon combustion.^[57]^ To comply with ASTM D7566 specifications, jet fuel must contain a minimum of 8 vol % aromatics to maintain engine seals through o‐ring swelling.[^57^, ^58^, ^59^] The SAF produced in this study may fulfill this requirement while providing improved combustion characteristics over existing SAF pathways, which predominantly yield paraffin and iso‐paraffin compounds. For the first time, this work presents a transformative approach for biorefinery by‐products containing high concentrations of PAHs, enabling the parallel production of biographite and SAF. By establishing a scalable, drop‐in pathway for the co‐production of these two critical products, this study contributes to advancing sustainable solutions for both the battery and aviation industries, reducing reliance on fossil‐derived resources and strengthening the circular economy of biomass‐derived feedstocks.

## Materials and Methods

### Materials

The ICCP bio‐oil used as the feedstock for this study was received from BioBTX, Groningen, Netherlands and was produced using a crude glycerol by‐product from biodiesel production. Two reference materials were used for comparison to the produced biographite: a commercially available calcined petroleum coke, or needle coke, and BCG18 synthetic graphite from Birla Carbon U.S.A., Inc., referred to herein as needle coke and commercial synthetic graphite, respectively. The needle coke reference was graphitized under identical experimental conditions (temperature, time, milling, etc.) as the biographite described herein to represent a commercially relevant reference and the resulting graphite will be referred to as needle coke graphite.

### Coking, Calcination, and Graphitization

The ICCP residual oil was heated in a round bottom flask for five hours at 500 °C to produce solid green bio‐coke. The condensate produced during coking (referred to herein as coke oil) was collected and characterized before being used as the feedstock for hydrotreating to fuels. Gas produced during coking is not captured or characterized, and is considered a loss. Calcination of the green bio‐coke was performed under nitrogen gas in a MTI OTF‐1200 C tube furnace equipped with a Kanthal APM alloy tube at 1000 °C for four hours with a heating rate of 5 °C min^−1^. The tube furnace was then allowed to cool to room temperature. Lastly, the calcined bio‐coke was graphitized under helium gas in a Thermal Technologies LLC 668 G resistive‐heated graphite furnace at 2800 °C, in which the temperature was ramped at 25 °C min^−1^ to 2500 °C and then ramped at 3 °C min^−1^ to 2800 °C, holding at 2800 °C for one hour. The graphite furnace was then allowed to cool to room temperature. The calcined needle coke reference was graphitized under identical parameters. Following graphitization, both materials were jet milled prior to battery testing.

### Gel‐Permeation Chromatography

The molecular weight distribution or the ICCP oil and Coke oil were measured by gel permeation chromatography (GPC). Samples (50 mg) were dissolved in 50 mL of tetrahydrofuran (THF, no inhibitor). The samples were then filtered through a 0.45um nylon membrane syringe filter prior GPC analysis. GPC analysis was performed using an Agilent HPLC (1260 Infinity) with three GPC columns (Polymer Laboratories, 300×7.5 mm) packed with polystyrene‐divinylbenzene copolymer gel (10 um beads) having nominal pore diameters of 104, 103 and 102 Å, respectively. THF was used as the mobile phase and the flow rate was set to 1.0 mL min^−1^, and an injection volume of 25 μL was used. A diode‐array detector measuring absorbance at 260 nm (bandwidth 40 nm) was used for retention time detection. Retention time was converted into molecular weight with a calibration curve established using 18 polystyrene standards of known molecular weight, ranging from 580–980,000 g mol^−1^, and toluene was used to calibrate for low mass compounds (92 g mol^−1^). The molecular weights calculated are not absolute molecular weights but are an approximation based on the polystyrene standards. The GPC data was normalized to the total response for each sample prior to calculations and plotting.

### Fourier Transform Ion Cyclotron Resonance Mass Spectrometry

The ICCP oil and coke oil were analyzed by Fourier transform ion cyclotron resonance mass spectrometry (FT‐ICR MS) with chemical ionization. The data was collected with a Bruker Solarix 7 T FT‐ICR MS. Each sample was dissolved in acetone at a concentration of 10 mg Ml^<1^ then further diluted in methanol to a final concentration of 150 μg mL^−1^ prior to analysis. The diluted samples were directly infused at a flow rate of 20 μL min^−1^, and 25 individual spectra were co‐added to produce the final averaged spectrum. Atmospheric pressure chemical ionization (APCI) was used based on previous work with the following conditions: Capillary voltage of 4500 V, endplate offset of −500 V, nebulizing gas pressure of 2 bar, dry gas temperature of 200 °C, desolvation gas flowrate of 2.5 L min−1, corona of 4500 nA, low m/z cutoff of m/z 100, and time‐of‐flight of 0.7 s.^[60]^ The data was exported to CSV and chemical formula assignments were generated with PetroOrg software. The plotting of FT‐ICR MS data was performed in Microsoft Excel and R software.

### Gas Chromatography Mass Spectroscopy with PolyArc‐FID

Compositional analysis of the ICCP oil and coke‐oil by gas chromatography coupled with mass spectrometry was performed on an Agilent 8890 Gas Chromatograph with 8977B mass selective detector (MSD) with a PolyArc‐FID. The GC was equipped with a post column flow splitter for simultaneous MS‐FID analysis. A PolyArc detector was placed in line with the FID for quantitation and the response was verified with a mixture of representative compounds. The PolyArc system acts as a methanizer, converting all organic components to methane and generating detector response relative to carbon number. Samples were diluted 1 : 10 gravimetrically in acetone. The injection volume was 1 μL and the split ratio was 1 : 100. The inlet temperature was 275 °C. The column used for compound separation was a 30 m x 0.25 mm x 0.25 μm Restek Rtx‐50 (50 %‐phenyl‐methylpolysiloxane phase). The oven temperature was held at 35 °C for 2 min, then increased to 300 °C at a heating rate of 5 °C min^−1^ and then held at 300 °C for 10 min. The MSD was operated in continuous scan mode from m/z 29 to 300. Both FID and MSD transfer lines were set to 350 °C.

Analytes identified via GC‐MS‐Polyarc‐FID were quantified based on FID peak areas using response factors (Rf) relative to nonane (C_9_). External calibrations and calibration validation standards were also analyzed for validation purposes and for determination of response factors and adjustment factors if necessary relative to nonane. Example calculation for quantification of a specific analyte as mass % present in the sample is shown below (Eqiuation(1–3):

(1) $$Mass{\%}_{analyte}=FID\;Area_{analyte}\times Rf_{analyte}\times DF$$

(2) $$Rf_{analyte}=\;wt\%\;C_{9\;standard}\times \frac{9}{C{\#}_{analyte}}\times \frac{MW_{analyte}}{128}\times \frac{1}{Area_{C_{9\;standard}}}$$

(3) $$DF\;\left(dilution\;factor\right)=\;\frac{mass\;oil\;sample+mass\;solvent}{mass\;oil\;sample}$$

### Thermogravimetric Analysis

Thermogravimetric analysis (TGA) was performed using a TA Instruments Discovery Thermogravimetric Analyzer on the biographite to measure ash content and deduce carbon content of the material. The samples were loaded into the sample holder and heated at a rate of 10 °C min^−1^ until a maximum temperature of 800 °C under nitrogen gas. Once the maximum temperature was met, the atmosphere was switched to air and held for 60 minutes.

### X‐Ray Diffraction

X‐ray diffractograms of the solid carbon materials were taken on a PANalytical X'Pert Pro X‐Ray Diffractometer equipped with a copper‐Kα radiation source (wavelength set to 1.5418 Å) and an X'Celerator Detector. Diffraction patterns were obtained using a scan rate of 5° two‐theta per minute. (002) d‐spacing and crystallite size were calculated using the Scherrer equation (Eqaution (4)) and Bragg's Law (Equation (5)) as described by Lee *et al*. where L is the crystallite size, K is the shape factor constant, λ is the X‐ray wavelength, B is the width of the peak at the half‐height, θ is the Bragg angle of the beam, and d is the interplanar spacing.^[61]^

(4) $$L=\;\frac{K\lambda }{Bcos\left(\theta \right)}$$

(5) $$d=\;\frac{\lambda }{2sin\left(\theta \right)}$$

### Raman Spectroscopy

Raman spectra of the solid carbon materials were obtained using a Metrohm i‐Raman Prime spectrometer at a wavelength of 532 nm. Data acquisition and analysis was conducted using BWSpec® 4 with an integration time of 120 seconds. The extent of graphitization (α) was calculated from the Raman spectra of the calcined bio‐coke and biographite samples using the intensities of the *G* and *D* peaks (Equation (6)), represented as I_*G*_ and I_*D*_, respectively.^[48]^

(6) $$\alpha =\;\frac{I_{G}}{I_{D}+I_{G}}\times 100$$

### Scanning Electron Microscopy

A small amount of each solid carbon sample was dispersed onto carbon tape affixed to 12.5 mm aluminum pin mount sample stubs. The sample stage was then inserted into a JEOL JSM‐IT510 Scanning Electron Microscope (SEM). Images were acquired in standard, high vacuum mode using a 5 keV beam and a secondary electron detector (Everhart‐Thornley).

### Scanning Transmission Electron Microscopy

A Tecnai F20 Scanning Transmission Electron Microscope (S/TEM) instrument with accelerating voltage of 200 kV was used to analyze solid carbon samples. Samples were prepared by mixing a small volume of sample powder with tetrahydrofuran (THF), ultrasonicating the solution for 3 minutes, and depositing a small aliquot onto a copper TEM grid coated with lacy carbon film.

### Half‐Cell Electrode Preparation, Cell Assembly, and Electrochemical Characterization

The biographite, needle coke graphite, and commercial synthetic graphite were tested in CR2032 coin cells with the following electrode slurry: active graphite material (94.7 wt %), Conductex™ il4 carbon black (2 wt %), and carboxymethylcellulose (CMC):styrene butadiene (SBR) binder (ratio 1 : 2 by weight) (3.3 wt %) with water as the solvent and a high‐speed disperser (HSD, Dispermat). The slurry was cast onto copper foil current collector at a wet film thickness of 150 micron and dried in a convection oven at 80 °C for two hours before being dried at 60 °C under vacuum to remove the solvent. The dried electrodes were pressed to an active material loading density of 1.5 g cm^−3^ using a two‐roll mill. Electrodes with a 12‐mm diameter were punched and five coin cells from each graphite active material were assembled. The active material areal mass of the electrodes was typically 4–6 mg cm^−2^. Coin cells were assembled in an argon‐filled glove box. Lithium metal was used as the reference and counter electrode and the electrolyte consisted of 1.2 M lithium hexafluorophosphate (LiPF_6_) in ethylene carbonate (EC):ethyl methyl carbonate (EMC) solvent (3 : 7 by volume) with vinylene carbonate (VC) (2 wt %) and 1, 3‐propane sultone (PS) (1 wt %). Celgard 2325 separators were used. Cells were rested after assembly for 24 hours. Formation cycling was conducted using an Arbin battery cycler at room temperature. Galvanostatic charge‐discharge tests were performed in a voltage range of 0.01 to 1.5 V (vs. Li/Li^+^) at a current rate of C/20 (where 1 C corresponds to 335 mAh g^−1^) for four cycles.

### Full‐Cell Electrode Preparation, Cell Assembly, And Electrochemical Characterization

The biographite and commercial synthetic graphite reference were used for full‐cell configurations. The graphite anodes were paired with lithium nickel manganese cobalt oxide (NMC532) cathode in CR2032 full cell coin cells. The cathode materials were prepared with the following electrode slurry: NMC532 (96 wt %), Conductex™ il4 carbon black (2 wt %), polyvinylidene fluoride (PVDF) binder (2 wt %) with n‐methyl pyrrolidone (NM) as the solvent and mixed using a high‐speed disperser (HSD, Dispermat). The slurry was then cast onto aluminum foil current collector at a wet film thickness of 70 micron and the electrode was dried first in a convection oven for 2 hours at 80 °C before being completely dried in a vacuum oven at 60 °C. The dried electrodes were pressed to an active material density of 2.8 gcm^−3^ using a two‐roll mill. Discs with a 12‐mm diameter were punched for each cathode and 15‐mm diameter for each anode (using the preparation described for the half‐cell configuration) to assemble 5 full cells. The N/P ratio of the full cells was typically between 1.1–1.2. The electrolyte used in each cell was 1.2 M lithium hexafluorophosphate (LiPF_6_) salt dissolved in ethylene carbonate (EC) and ethyl methyl carbonate (EMC) solvent mixture with a ratio of 3 : 7 by volume. Vinylene carbonate (VC) (2 wt %) and 1,3‐propane sultone (PS) (1 wt %) were used as additives. A Celgard 2325 separator was used and the coin cells were assembled in an argon‐filled glovebox. The full cells were rested for 24 hours before being cycled on an Arbin battery cycler at room temperature. The cycling consisted of four charge/discharge cycles at C/20 (where 1 C is 150 mAh g^−1^ NMC532) with a constant voltage until the current decreased below C/100. The voltage window applied was 3.0 to 4.2 V vs. Li/Li^+^. After formation cycling, the full cells were cycled using a profile to test the rate capability. The profile consisted of five cycles of symmetric, constant current cycling at C/10, C/5, C/3, C/2, 1 C, and 2 C. The current was then returned to C/10 for the remaining cycles as a health check.

### Hydrotreatment of Coke Oil

The coke oil was hydrotreated in a continuous 122‐cm tall trickle bed reactor with an inner diameter of 8.4 mm. A six‐point thermocouple with an outer diameter of 3.2 mm was placed along the centerline, and the catalyst was loaded in the annulus between the thermocouple and reactor wall. The reactor tube was surrounded by a bronze sleeve to enhance heat transfer to the reactor. The reactor assembly was placed in a four‐zone furnace. For these experiments, the temperature in the bottom sections was set to 385 °C, and the temperature in the top sections increased gradually from 180 °C to 385 °C. The operating pressure was 125 bar (1800 psi). The oil was fed into the reactor via an ISCO pump, and compressed H_2_ with 60 ppm H_2_S was fed via a mass flow controller. The reactor products were condensed in vessels placed in a chilled bath at 5 °C, and any remaining liquids were collected in a condenser chilled to −5 °C. The pressure was controlled by a back‐pressure regulator, and the exit gas flow rate was measured by a Coriolis flow meter. The exit gas was analyzed by an online micro gas chromatograph for C_1_‐C_5_ hydrocarbons, carbon oxides, and hydrogen. The catalyst was a commercial NiMo/Al_2_O_3_ catalyst (Topsoe TK‐431), crushed and sieved to a particle size 0.25–0.5 mm. 16 g of the catalyst and 32 g of SiC (Green Silicon Carbide, 24 grit from Panadyne) were placed in the transition zone from ~180 °C to 385 °C and 12.5 g of catalyst in the isothermal zone at 385 °C. The coke oil feed rate was 2.5 mL h^−1^ and the feed rate of the H_2_/H_2_S mixture was 175 smL min^−1^ (standard ml min^−1^), resulting in a H_2_:oil ratio of 4,200 sL L^−1^. The total weight hourly space velocity (WHSV) was 0.1 g (g catalyst h) ^−1^. The catalyst was presulfided in the reactor using 35 wt % di‐*tert*‐butyl disulfide in decane, according to the following procedure: heat to 150 °C in 165 smL min^−1^ of the H2/H_2_S mixture at 2.5 °C min^−1^, start sulfiding liquid flow at 0.055 mL h^−1^, hold for 2 hours, and ramp to 385 °C at 1.5 °C min^−1^, hold for 4 hours. The hydrotreating experiment was continued for 111 hours, and the liquid products after an initial 21‐hour stabilization period were collected. The liquids separated into an organic hydrocarbon product and an aqueous phase. The combined organic product was distilled in a B/R 800 micro spinning band distillation, first by atmospheric distillation, and then at 0.066 bar vacuum (50 torr) as described elsewhere.[^62^, ^63^]

### SAF Characterization

The combined hydrotreated product and the SAF fraction were analyzed for ultimate composition at Huffman Hazen Laboratories (Golden, CO), for direct oxygen by an Elementar VL Cube with ALS Autosampler. The fraction boiling in the SAF range was analyzed for simulated distillation according to ASTM D2887, flash point according to ASTM D6450, freeze point according to ASTM D2386, heating value according to ASTM D1405, density according to ASTM D4052, kinematic viscosity according to D4052, surface tension according to ASTM D1331, and indicated cetane number (ICN) according to ASTM D8183.[^64^, ^65^, ^66^, ^67^, ^68^, ^69^, ^70^]

### Two‐Dimensional Gas Chromatography

Two‐dimensional gas chromatography with time‐of‐flight mass spectrometry and flame ionization detection (GC x GC ‐ TOF MS ‐ FID) was used to determine the composition and quality of the hydrotreated coke oil and SAF fraction with an Agilent 8890 GC (Agilent Technologies, Palo Alto, CA, USA) equipped with a flame ionization detector (FID) connected to a Bench TOF 2 time of flight mass spectrometer (Markes International, Bridgend, UK).[^71^, ^72^, ^73^] The primary column was a BPX5 (20 m×0.18 mm×0.18um, SGE Analytical Science) and the secondary column was a BPX50 (5 m×0.25 mm×0.10um, SGE Analytical Science). The system was equipped with a multimode inlet and an Insight flow modulator (SepSolve Analytical, Ontario, Canada). The modulation period was set to 4 s with a flush time of 300 ms. A 0.2uL neat sample was injected at 250 °C with a 300 : 1 split ratio. The carrier gas through the system was ultra‐high purity helium. The oven temperature was started at 40 °C, raised by 1.5 °C min^−1^ to 280 °C, and held for 2 minutes. Eluted compounds were transferred to the time‐of‐flight mass spectrometer (TOF‐MS) via the transfer line and ion source, operated at 260 °C and 280 °C, respectively. The TOF‐MS scanned scan range was 29–600mu at a data rate of 50 spectra s^−1^. Eluted compounds were also analyzed by flame ionization detector (FID), which was at a temperature of 300 °C with hydrogen flowing at 40 mL min^−1^. The samples were analyzed using ChromSpace GC x GC software (SepSolve Analytical, Ontario, Canada) with the TOF‐MS and FID responses. All quantitative analyses were based off the FID responses using effective carbon number relative response factors to nonane.

External calibrations and calibration validation standards were also analyzed by the GC x GC‐ TOF MS – FID for validation purposes and for determination of response factors relative to nonane. Compound classes were validated based on mass spectra and retention times from external standards and quantified in samples by integrating peaks in regions corresponding to appropriate compound classes. Example calculations for quantification of a specific analyte and/or compound class as wt % present in the sample is shown below (Equation (7–11)) with ECN contributions listed in Table 1:

(7) $$Wt\;\%=FID\;Area\;\times Rf_{analyte}$$

(8) $$Rf_{analyte}=RRf_{analyte}\;\times Rf_{C_{9}}$$

(9) $$RRf_{analyte}=\;\frac{MW\times 9}{128\;\times ECN}$$

(10) ECN=∑Carbon#×ECNcontribution

(11) $$Rf_{C_{9}}=\frac{C_{9}\;wt\%\;in\;standard}{C_{9}\;area\;in\;standard}$$

**Table 1:** Compound classes and associated ECN contributions used to quantify compounds detected via two‐dimensional gas chromatography with time‐of‐flight mass spectrometry and flame ionization detection (GC x GC – TOF MS – FID).

Atom	Type	ECN Contribution
C	Aliphatic	1
C	Aromatic	1
C	Olefinic	1
C	Acetylenic	1.3
C	Carbonyl	−1
C	Carboxyl	−1
C	Ether	−1
C	Primary alcohol	−0.5
C	Secondary alcohol	−0.75
C	Tertiary alcohol	−0.25

## Results and Discussion

### Coking ICCP Bio‐Oil

**Figure 2:**
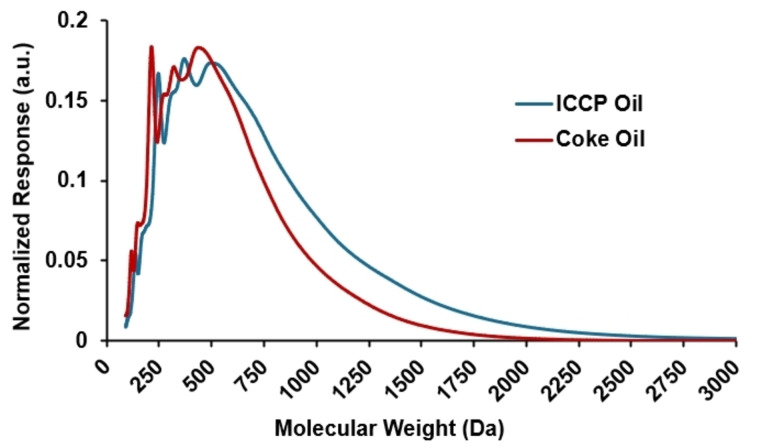
Gel permeation chromatography (GPC) profiles of untreated ICCP oil and coke oil obtained via distillation of vapors from coking at 500 °C.

**Figure 3:**
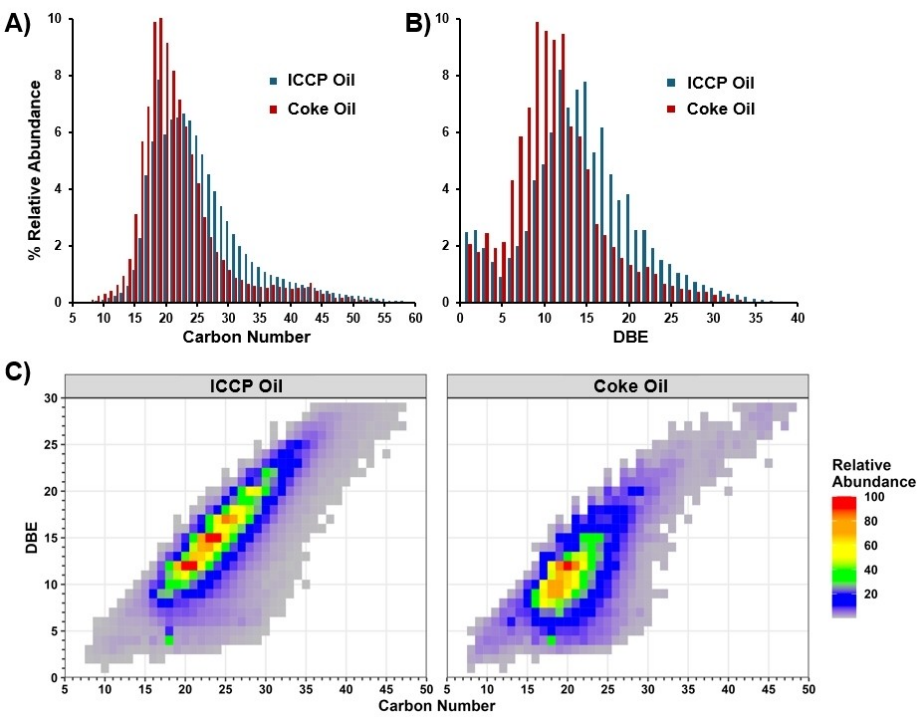
FT‐ ICR MS data including positive‐ion APCI derived (A) carbon distribution, (B) double bond equivalent (DBE) distribution, and (C) carbon number vs DBE of ICCP oil and coke oil.

The ICCP bio‐oil was coked at 500 °C, which yielded 19.6 wt % green bio‐coke (solid) and 78.2 wt % coke oil (liquid). The high yield of oil is due to the high composition of mid‐range carbons that readily volatilize before coking reactions can occur. The ICCP oil has a low oxygen content (<3 wt %) which leads to reduced polymerization and coke yields. To determine the effects of coking on oil composition, the ICCP oil and coke oil were analyzed by GPC and FT‐ICR MS. The GPC chromatograms in Figure 2 show that the ICCP oil (blue) is composed of greater concentrations of compounds with apparent molecular weights greater than 500 Da. The coke oil, however, shows reduced concentrations in the compounds above 500 Da, which indicates that the largest compounds in the ICCP oil are removed due to the formation of coke. This is to be expected, as the least volatile compounds remain in the reactor and are allowed to polymerize to form coke (referred to herein as green bio‐coke). The same trend is observed with FT‐ICR MS, where Figure 3A shows an increased abundance in the ICCP oil compounds with greater than 24 carbon atoms relative to the coke oil, which shows increased abundance in compounds ranging from C_15_‐C_23_. Figure 3B plots the distribution of double bond equivalents (DBE=C‐(H/2) + (N/2) + 1) for the ICCP and coke oils, which correspond to the degree of aromaticity for these oils. The double bond equivalent (DBE) distribution reveals that the compounds with high DBE are enriched in the ICCP oil and decrease in abundance after coking. The trend is also observed in Figure 3C–D which plots the carbon number vs. DBE values for each oil and shows a shift to lower carbon and DBE values after coking. The molecular trends observed via GPC and FT‐ICR MS are consistent with the reactions observed during the delayed coking of petroleum feeds, wherein, larger PAHs, which are less volatile and can more easily stabilize radical structures, condense and fuse together to form the solid high‐molecular weight coke product, and lighter aromatic compounds, including phenols and aromatics, are fractioned off.^[54]^

**Figure 4:**
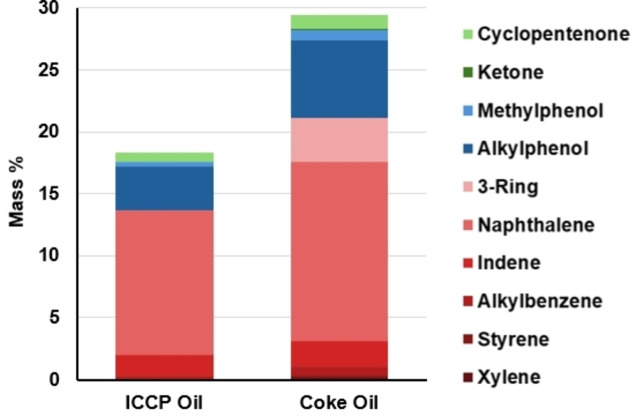
GC‐MS analysis of ICCP oil and coke oil by compound wt % where red, blue, and green shades correspond with aromatic, phenol, and carbonyl compound groups, respectively.

The compositions of the ICCP oil and coke oil were further elucidated via GC‐MS‐Polyarc‐FID, visualized in Figure 4 where red, blue, and green shades correspond with aromatic, phenol, and carbonyl compound groups, respectively. GC‐MS is ideal for determining the chemical composition of the low‐molecular weight volatile compounds in bio‐oils; however, higher molecular weight compounds with low volatility are not observed by GC‐MS. The results in Figure 4 indicate that 18.3 and 25.9 wt % of the ICCP and coke oils, respectively, are detectable by GC‐MS. The increase in the fraction of volatile compounds that are quantified by the GC‐MS method indicates that heavy or high‐molecular weight compounds were removed from the oil stream during coking due to the formation of coke. This agrees with the GPC and FT‐ICR MS results shown above.

### Graphitization

**Figure 5:**
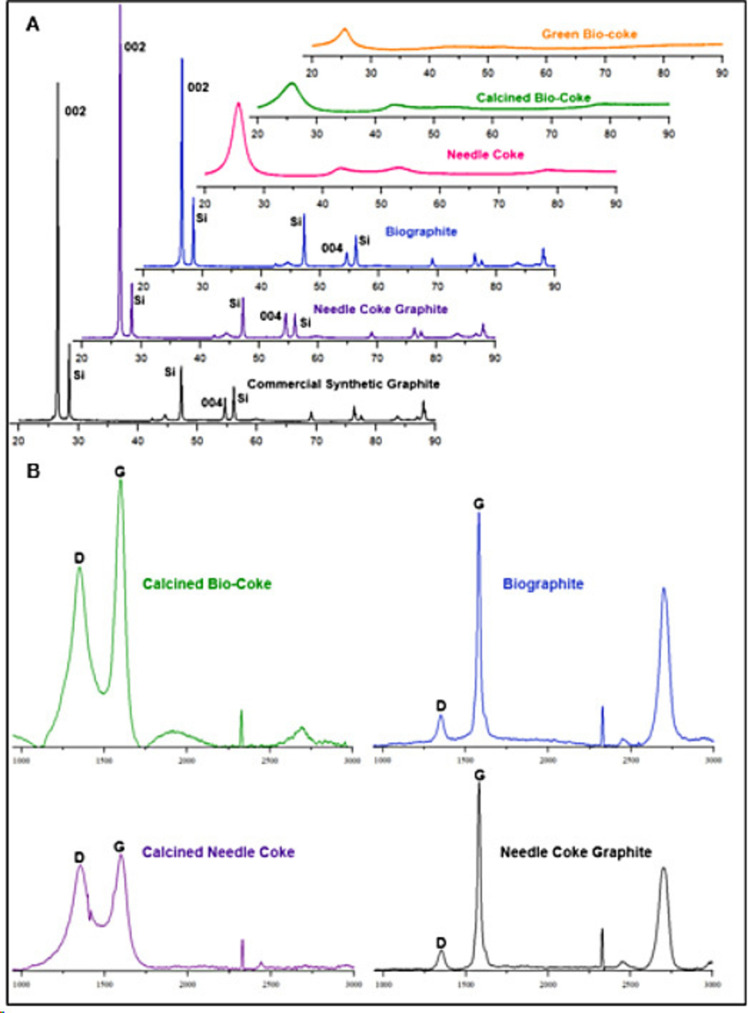
(A) X‐ray diffractograms of green bio‐coke, calcined bio‐coke, biographite, needle coke graphite, and commercial synthetic graphite with 2θ angles of diffraction on the x‐axis. Silicon was used as a standard and the correlated peaks are labeled as such. (B) Raman spectra of calcined bio‐coke, biographite, calcined needle coke, and needle coke graphite with chemical shift on x‐axis.

X‐ray diffraction (XRD) and Raman spectroscopy are the two most common methods used to quantify and qualify graphite crystallinity and as such, are used to estimate graphite quality. A sharp peak at the 2θ diffraction angle of 26.5° is characteristic of the graphite (002) reflection.[^32^, ^40^, ^48^] For comparison to fossil fuel‐derived products, a petroleum‐based needle coke was graphitized in parallel to the calcined bio‐coke, and a high‐quality commercially available synthetic graphite reference material was also analyzed. The XRD patterns throughout the coking, calcination, and graphitization of the ICCP oil feedstock (and graphitization of the calcined needle coke reference) show the development of graphite as evident by the sharpening of the (002) peak, corresponding to an increase in crystallite size and decrease in d‐spacing (Figure 5A).^[61]^ Silicon was used as an internal standard and the associated peaks can be observed in the graphite XRD patterns^[74]^. As shown in Table 2, the d‐spacing, or interlayer spacing, calculated using Bragg's Law, of the graphene sheets for the three graphite samples were 3.365, 3.365, and 3.359 Å for the biographite, needle coke graphite, and commercial synthetic graphite samples, respectively. These values are in agreement with the consistently reported spacing of pristine graphite (3.36 Å).^[75]^ Using the XRD patterns and the Scherrer equation, the average graphitic crystallite size in the stacking or *c*‐direction (L_*c*_) and plane width (L_*a*_) are determined to be 45.1 and 10.4 nm, 43.0 and 11.7 nm, and 45.8 and 29.6 nm for the biographite, needle coke graphite, and commercial synthetic graphite samples, respectively, as shown in Table 2. The measurement of the biographite that can be attributed to the height of the graphene sheets or number of layers agglomerated (L_c_) is comparable to the needle coke graphite and commercial synthetic graphite references. Raman spectroscopy is used to identify the two primary Raman shifts characteristic of graphite: the smaller intensity D shift (at ~1350 cm^−1^) and the larger intensity G shift (at ~1575 cm^−1^) (Figure 5B). The ratio of the D and G shift intensities is used to determine the extent of graphitization, α, from Equation (3),[^40^, ^48^] with values presented in Table 2. The degree of graphitization of the bio‐based materials increased from 59.3 % to 87.3 % upon graphitization. Comparatively, the degree of graphitization of the needle coke, that underwent the same graphitization procedure, increased from 51.8 % to 90.3 %. Raman spectra of commercial synthetic graphite reported an extent of graphitization of 92.0 %. Thus, the biographite supported a similarly uniform graphitization, with slightly more areas of disorder than the petroleum‐based references.

**Table 2:** Summary of results from XRD and Raman characterization of green bio‐coke, calcined bio‐coke, calcined needle coke, biographite, needle coke graphite, and commercial synthetic graphite.

	Green Bio‐coke	Calcined Bio‐coke	Calcined Needle Coke	Biographite	Needle Coke Graphite	Commercial Synthetic Graphite
d spacing (nm)	0.34824	0.34399	0.34533	0.33650	0.33650	0.33591
L_c_ (nm)	3.1	2.1	3.9	45.1	43.0	45.8
L_a_	‐	‐	‐	10.4	11.7	29.6
α (%)	‐	59.3	51.8	87.3	90.3	92.0

**Figure 6:**
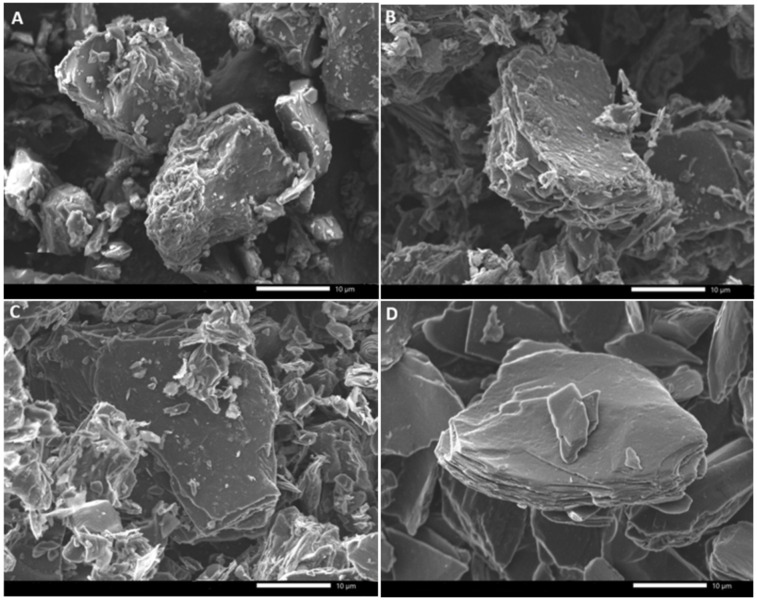
Scanning electron microscopy images of (A) green bio‐coke, (B) calcined bio‐coke, (C) biographite, and (D) commercial synthetic graphite.

SEM images of the green bio‐coke, calcined bio‐coke, and biographite demonstrate the formation of flattened, layered agglomerates consistent with the morphology of commercial synthetic graphite (Figure 6). During the three‐step heat treatment (Figure 6A−C) there is an observable reduction in disordered “fuzzy” carbon particles as the carbon material graphitizes. Layered stacking of platelets can be observed after the calcination step (Figure 6B) and these sheets become larger after the final graphitization step (Figure 6C). Processing post‐graphitization, such as sieving and shaping, could improve the appearance of the biographite (Figure 6D).

**Figure 7:**
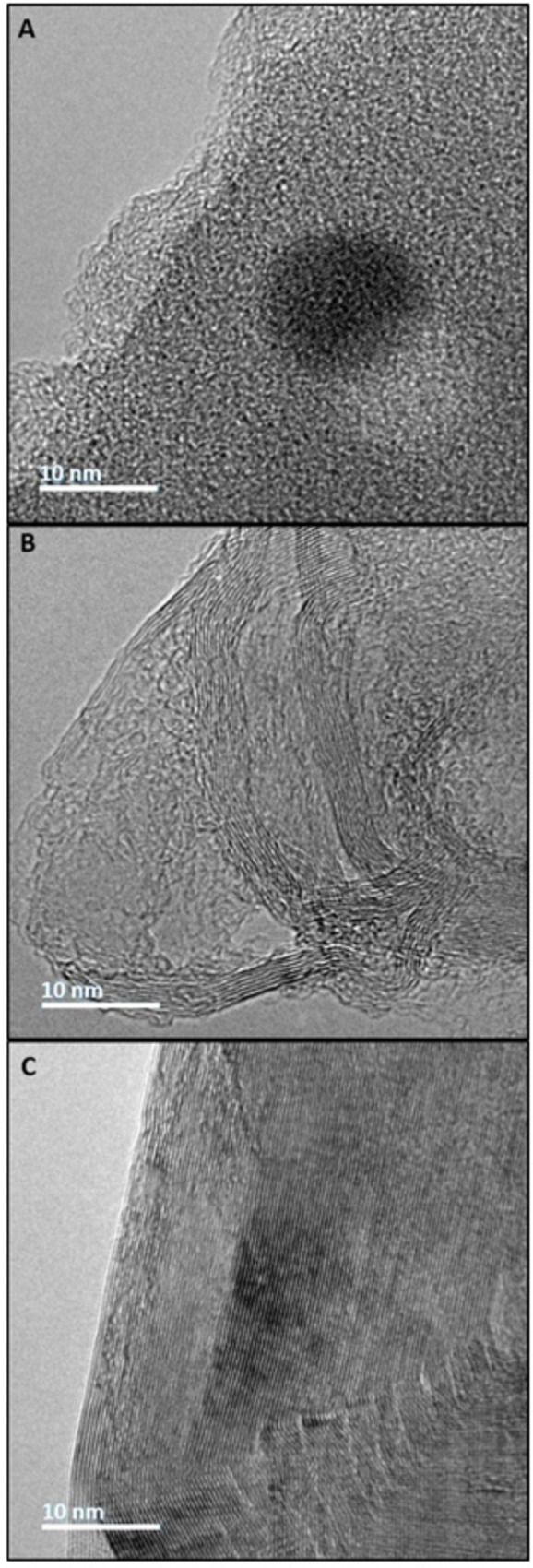
Scanning transmission electron microscopy (S‐TEM) images of (A) green bio‐coke, (B) calcined bio‐coke, and (C) biographite.

Scanning transmission electron microscopy (S‐TEM) images were taken to visualize the morphology and evolution of the biographite through the three‐stage pyrolytic process from disordered green bio‐coke to more turbostratic calcined bio‐coke, finally to ordered crystalline biographite (Figure 7). The formation of graphitic sheets was observed in the calcined bio‐coke sample, and the stacking height in the *c*‐direction (L_*c*_) dramatically increased upon final graphitization with a L_*c*_ of >40 nm, which corroborates the average L_*c*_ of the biographite determined via XRD (Table 2).

Thermogravimetric analysis (TGA) was conducted to determine the carbon content of the biographite produced from ICCP bio‐oil. The analysis revealed an ash content of 0.017 wt %, indicating minimal inorganic impurities. Typically, the carbon content of graphite is estimated by assuming that carbon and ash are the primary components. Based on this assumption, the biographite exhibited a calculated carbon content of 99.983 wt %, which is comparable to high‐purity synthetic graphite used in industrial applications.^[76]^ The high carbon content of the biographite, coupled with the comparable XRD and Raman Spectroscopy results suggests that biographite derived from ICCP bio‐oil could serve as a viable alternative feedstock for commercial synthetic graphite, offering a more sustainable production route.

### Electrochemical Testing

Critical parameters in electrochemical performance testing of graphite materials for use in lithium‐ion applications are specific capacity and Coulombic efficiency (CE). These metrics represent baseline parameters for electrochemical performance consideration and are the standard in most biographite studies.^[29]^ Additional qualities such as size, shape, packing efficiency, surface area, and purity can affect battery performance, but are not often the focus of proof‐of‐concept biographite studies. Traditionally, biographite research that does pursue electrochemical performance analysis utilizes half‐cell configurations that pair the biographite active material with a lithium metal reference and counter electrode. Half‐cell systems provide valuable information regarding the specific capacity and CE of experimental active material with a large lithium cation inventory provided by the counter electrode.^[77]^ The majority of biographite studies do not include full‐cell assembly electrochemical performance testing, which is not ideal given that full‐cell performance describes important parameters of the battery by pairing the anode with a commercially applicable cathode and observing the complete system rebound performance during increasingly fast cycling rates where the cathode does not have a theoretically unlimited inventory of lithium ions.^[29]^ Thus, an additional novelty of this study was the development and assessment of full‐cell battery configurations with rate/rebound evaluation.

**Figure 8:**
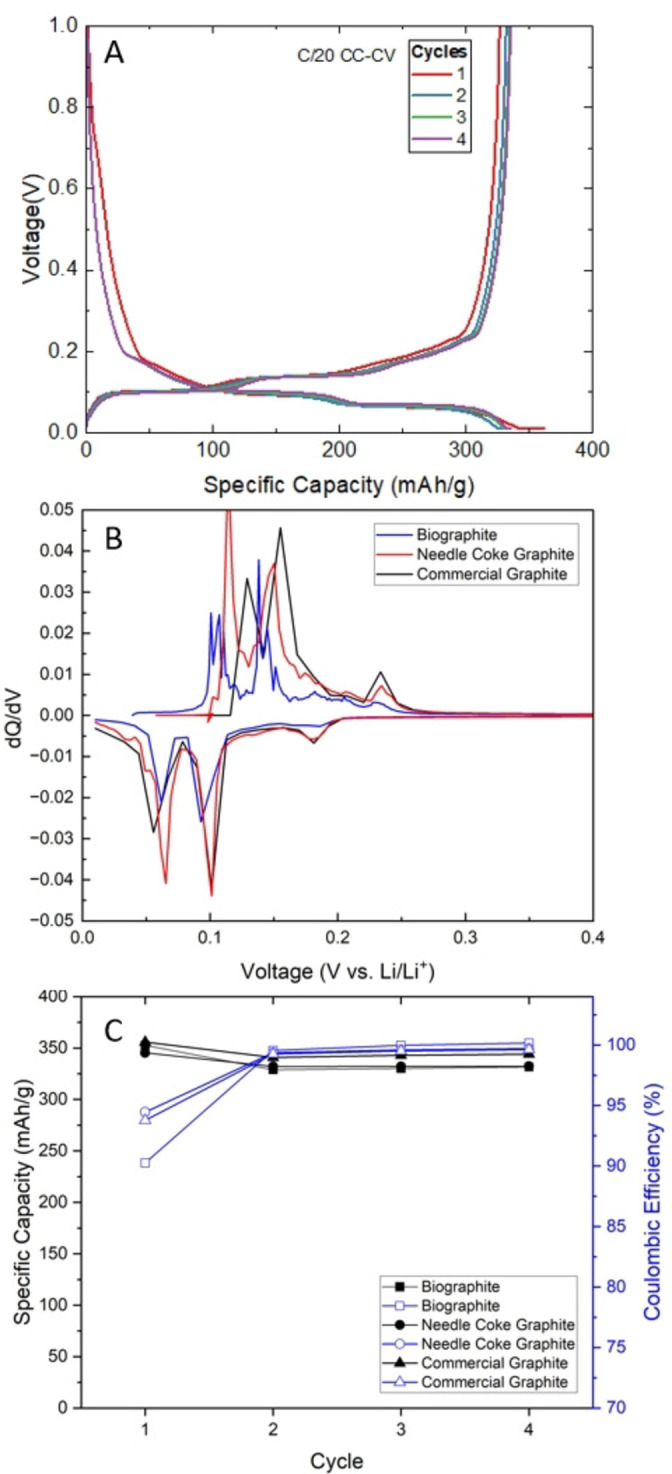
Electrochemical performance of biographite, needle coke graphite, and commercial synthetic graphite in half‐cell configurations. (A) Galvanostatic discharge and charge profiles of biographite half‐cells (B) Differential capacity plot (dQ/dV plot) for the fourth cycle of the biographite, needle coke graphite, and commercial synthetic graphite half‐cell batteries (C) Specific capacity of all half‐cell configurations in the first four cycles.

The performance of the biographite in the half‐cell configuration was similar to that of the reference graphite anodes with specific capacities of 332, 332, and 344 mAh g^−1^ for the biographite, needle coke graphite, and commercial synthetic graphite anodes, respectively (Figure 8). These values are comparable, and the agreement between the biographite and needle coke graphite specific capacities demonstrates the ability of the ICCP oil to be a drop‐in feedstock to the synthetic graphite production pathway. A galvanostatic charge discharge plot of the biographite half‐cell (Figure 8A) shows the expected profile and evolution of a solid electrolyte interface during the first charging cycle. Differential capacity plots of all anode material half‐cells (Figure 8B) display the anticipated shape of a typical graphite charging mechanism with no noticeable side‐reactions. The biographite anode had a first cycle CE (Figure 8C) of 90.7 %, which is in agreement with the target of >90 % for commercial lithium‐ion anode graphite.^[29]^ The first cycle CEs for the petroleum‐based coin cells were 94.4 % and 93.8 % for the needle coke graphite and commercial synthetic graphite, respectively. All cells exceeded a CE of 99 % in the second cycle and the final cycle CEs for the materials were 100 %, 99.7 %, and 99.6 % for the biographite, needle coke graphite and commercial synthetic graphite anodes, respectively. The higher CE of the petroleum‐based reference materials could be attributed to the materials’ higher degrees of graphitization (α) and lower impurities. When comparing with published literature on biographite materials made via graphitization with a catalyst, the biographite materials generated in this study without a catalyst perform better than most and equal to the best in terms of electrochemical performance including reversible capacity and CE.

**Figure 9:**
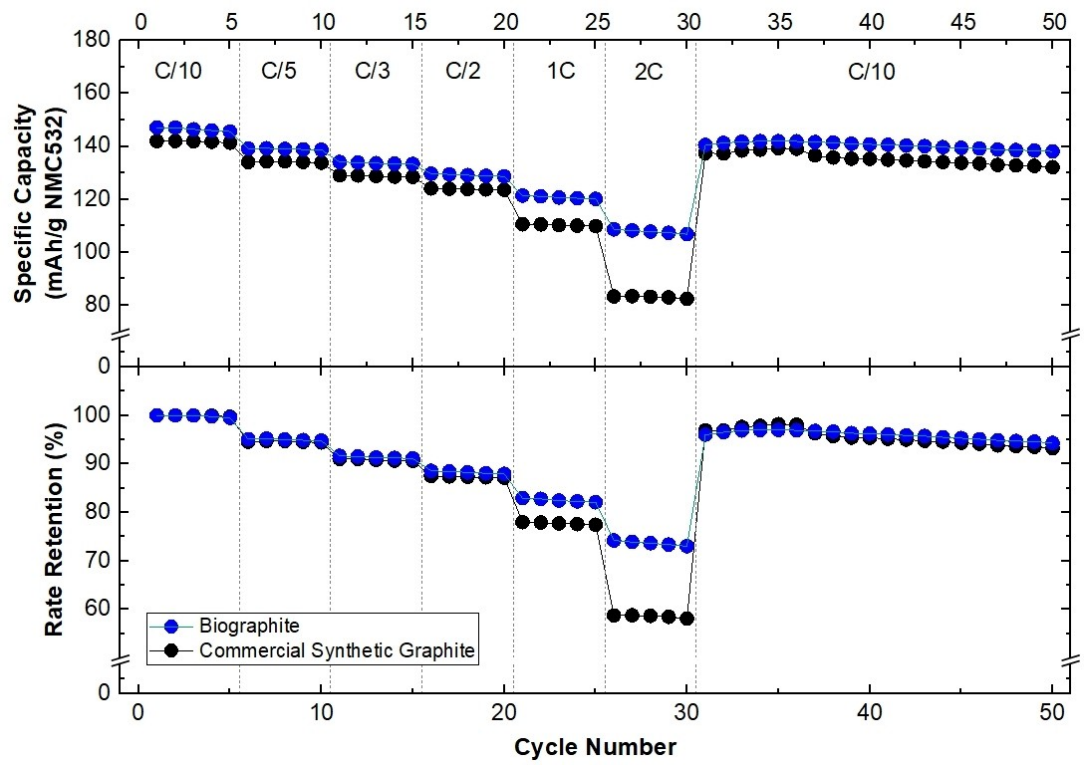
Electrochemical performance parameters including specific capacity (top) and rate retention (bottom) of full‐cell lithium‐ion batteries with biographite and commercial synthetic graphite anode materials and NMC cathode material. Rate capability current cycling at C/10, C/5, C/3, C/2, 1 C, and 2 C before returning to C/10, where C corresponds to 335 mAh g^−1^.

In response to the promising half‐cell specific capacity and initial CE results, full‐cells with a lithium nickel manganese cobalt oxide (NMC) cathode (opposed to half‐cells with Li metal) were constructed. Full‐cells were used for cycling rate tests to observe the battery's ability to rebound from increasing cycling rates (Figure 9). It should be noted that the full‐cell battery results are not used for specific capacity comparison, but only capacity rebound after current cycling rate increase and relaxation. The commercial synthetic graphite used in the full cell configurations had a different particle size distribution than the biographite, with Dv50 values of approximately 18 μm and 13 μm for the commercial synthetic graphite and biographite, respectively. Particle size distribution can have a significant impact on specific capacity and rate retention with larger particles (Dv50>15 μm) commonly used for higher energy density applications and smaller particles (Dv50<15 μm) commonly used for high rate/fast charging applications.^[78]^ Future work should investigate the effect of particle size on the performance of biographite, and other avenues that would optimize the electrochemical performance of the anode material. Regardless of the particle size distribution, the biographite supported a 96.8 % capacity rebound after high current cycling, comparable to the commercial synthetic graphite rebound of 96.7 %. The rebounding behavior of the biographite anode material should be further investigated for fast‐charging applications in the future.

### SAF Production

The condensed vapors from ICCP oil coking, referred to as coke oil, yielded a low oxygen and highly aromatic oil that can be upgraded to fuels, such as SAF, via hydrotreating. The coke oil was hydrotreated in a continuous hydrotreater over a commercial sulfided NiMo/Al_2_O_3_ catalyst to remove oxygen and hydrogenate double bonds. The successful hydrotreating of the coke oil indicates that the ICCP coke oil can be a drop‐in option for upgrading to renewable fuels in existing petroleum refineries. The hydrotreated product was further distilled into boiling point fractions that correspond to final transportation fuels. Table 3 shows the hydrotreating yields on a mass‐ and carbon‐basis. The organic product from hydrotreating comprised 93 % of the mass, whereas water only comprised 6 %. We also observed a good carbon balance with 97 % of the mass of carbon accounted for. The hydrotreated product was tested for elemental analysis and ash composition and showed a molar ratio of hydrogen:carbon of 1.8, with no measurable nitrogen, oxygen, sulfur, or ash. These characteristics show that ICCP‐derived coke oil is a good candidate for upgrading via hydrotreating.

**Table 3:** Summary of coke oil hydrotreating results.

			Mass yield, g/g feed		C yield, g C/g C in feed	
Organic			93 %±3 %		94 %±4 %	
Aqueous			6 %±1 %		‐	
Gases			2.0 %±0.0 %		1.5 %±0.2 %	
H_2_ consumed			4.1 %±0.4 %		‐	
Closure			95 %±3 %^[a]^		97 %±3 %	
Organic composition, wt %						
C	H	N	O	S	Ash	Water
87.25	13.50	<0.01	<0.01	<0.01	<0.01	<0.1

[a] g/(g feed + H_2_ consumed)

Table 4 shows the product distribution from distillation, where 70 % of the hydrotreated product distilled in the SAF range. Smaller amounts of gasoline‐ and diesel‐range distillates were also produced, 12 and 15 % respectively. The yield of the SAF fraction was significantly higher than the yields reported from hydrotreating of lignocellulosic‐derived catalytic fast pyrolysis oils (70 % vs. approximately 50 %).^[62]^

**Table 4:** Summary of distillation results (AET – Atmospheric Equivalent Temperature).

Fuel Fraction	AET, °C	wt %
Gasoline	<140	12 %
SAF	140–265	70 %
Diesel	265–300	15 %
Residue	>300	1 %
Losses		2 %

**Figure 10:**
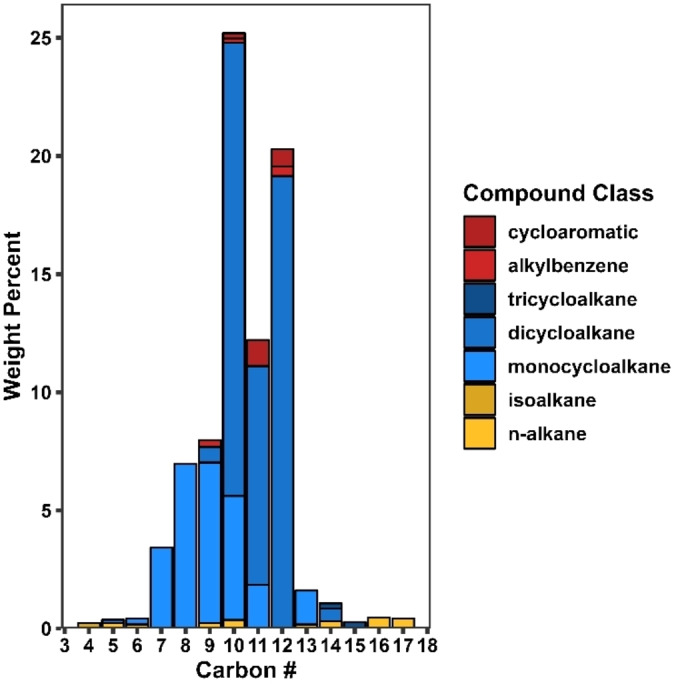
GC x GC ‐ MS derived carbon distribution for the SAF fraction of the hydrotreated and distilled coke oil where cycloalkanes are represented in blue shades, aromatics in red, and saturated alkanes in gold.

GC x GC‐MS was used to determine the composition and quality of the hydrotreated coke oil and SAF fraction. The ICCP coke oil was comprised primarily of aromatic hydrocarbons and phenols with multiple aromatic rings (Figure 4). However, after hydrotreating, GC x GC‐MS shows conversion to primarily cycloalkanes (82.5 %) and small amounts of *n*‐alkanes, iso‐alkanes, and aromatics (Table 5). Analysis of the SAF fraction revealed a similar composition, with the SAF comprised of 82.4 % cycloalkanes and a much smaller amount of *n*‐alkanes and aromatics, 1.1 and 3.9 %, respectively. This composition of fuel may be ideal for SAF due to the increased energy density of cycloalkanes compared to n‐alkanes and the reduced sooting of cycloalkanes compared to aromatics. There is also evidence that cycloalkanes may be a good replacement for aromatics with respect to seal swelling.^[79]^ Currently, no other commercial pathway to SAF is capable of producing cycloalkanes.^[80]^ Figure 10 shows the product distribution for the SAF fraction from GC x GC MS. The cycloalkanes are represented in blue shades, aromatics in red, and saturated alkanes in gold. The majority of cycloalkanes were comprised of mono‐ and di‐cycloalkanes. The carbon distribution of monocycloalkanes shows that the majority are observed at carbon numbers greater than C_7_, with a maximum at C_9_, which indicates the presence of alkylated cycloalkanes. A similar trend is observed for the dicycloalkanes, although to a lesser extent. The most abundant dicycloaklanes are present at C_10_, likely decalin, but significant amounts are also observed at C_11_ and C_12_. Monocycloalkanes have been shown previously to increase the energy density and specific energy of Jet A when added at 15 %, and dicycloalkanes were also shown to increase energy density^[81]^. However, the amount of dicycloalkanes also increases the overall density of the fuel, which can push the SAF properties out of ASTM specification ranges. Aromatic compounds in the SAF were observed primarily as cycloaromatics, which are attributed to incomplete hydrogenation. Alkanes were also present in small quantities, but no discernable trends were observed for these compounds.

**Table 5:** Summary of GC x GC ‐ MS analysis by compound group.

Compound Group	Hydrotreated Coke Oil (wt %)	SAF(wt %)
Aromatics	3.2	3.9
n‐Alkanes	2.6	1.1
Isoalkanes	0.3	0
Cycloalkanes	82.5	82.4
Total detected	88.6	87.4

^2^Data from GC x GC‐MS

The SAF fraction was tested for several key fuel properties (Table 6). The volatility by simulated distillation, freeze point, and flash point were all within the guidelines published in ASTM D4054. The fuel density was slightly above guideline values and the cetane number was slightly below guideline values. These properties are both due to the high concentration of cycloalkanes, but these shortcomings could be addressed by blending with more aliphatic fuel streams. This SAF stream, given that it has lower‐than‐acceptable cetane numbers, could serve as a compliment to other SAF pathways (HEFA, Fischer‐Tropsch, and Alcohol‐to‐Jet) that alone can only be used in blends with conventional jet fuel up to 50 vol%.^[80]^ The overall composition and yield of SAF from coke oil shows that the delayed coking pathway for ICCP oil is a viable option to produce SAF blend stock that may be used as a drop‐in source of aviation fuel while also producing a high‐value biographite co‐product.

**Table 6:** SAF fraction properties and guidelines from ASTM standards D4054 and D7566*.

Property	D4054/D7566*	Measured
ICN	35–60 (DCN)	29
Flash point, °C	38–66	56
Freeze point, °C	≤‐40	<‐75 °C
Carbon, wt %		86.79
Hydrogen, wt %		13.40
Nitrogen, wt %		<0.01
Oxygen (diff), wt %		<0.01
Sulfur, wt %	≤0.3*	<0.01
Ash, wt %		<0.01
Water (KF)*, wt %		<0.01
Oxygen (direct), wt %	C+H≥95*	<0.01
LHV, MJ kg^−1^	≥42.8	42.7
Viscosity –20 °C, mm^2^ s^−1^ K^−1^	≤8	5.5
Density at 15 °C, kg m^−3^	775–840	856
Surface Tension at 20 °C, mN m^−1^		27.9
D86 simdist		
T10	150–205	181
T50	165–229	198
T90	190–262	224
FBP	<300	247
T50‐T10	≥15	17
T90‐&T10	≥40	43

**Figure 11:**
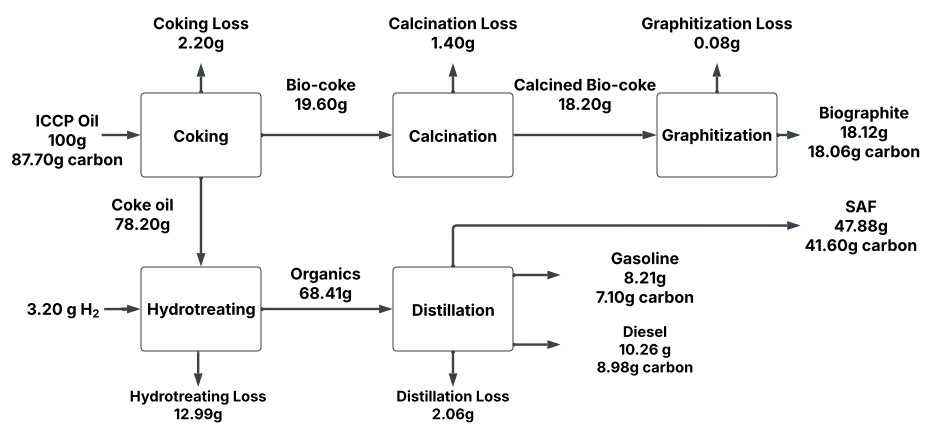
Schematic illustrating the mass and carbon balance of the process for converting PAH‐rich ICCP bio‐oil into biographite and SAF.

A schematic illustrating the mass and carbon balance of the process for converting PAH‐rich ICCP bio‐oil into biographite and SAF is shown in Figure 11. In this schematic, the wt % of the feedstock is simplified to show 100 g of ICCP bio‐oil (containing 87.70 g carbon) being fed into the coking process, yielding 19.60 g of solid bio‐coke and 78.20 g of coke oil. A coking loss of 2.20 g is attributed primarily to non‐condensable gaseous byproducts. The bio‐coke is subsequently calcined at 1000°C to remove residual volatile matter, resulting in 18.20 g of calcined bio‐coke and a 1.40 g calcination loss. Graphitization at 2800°C further reduces impurities, leaving 18.12 g of biographite and a 0.08 g graphitization loss. Notably, the biographite retains 18.06 g of carbon, indicating an extremely high carbon purity that was determined via TGA. Meanwhile, the 78.20 g of coke oil undergoes hydrotreating in the presence of hydrogen, wherein 3.20 g hydrogen is consumed. The hydrotreating step yields an organic fraction of 68.41 g suitable for fuel production, along with a 12.99 g loss (including gas and the aqueous fractions listed in Table 3). Subsequent distillation of the organic stream separates the hydrotreated organics into three main fractions: gasoline (8.21 g, 7.10 g carbon), diesel (10.26 g, 8.98 g carbon), and SAF (47.88 g, 41.60 g carbon) while incurring a 2.06 g distillation loss. The SAF fraction constitutes the majority of the liquid fuel product, corresponding to roughly 48 wt % of the total mass fed and 47 wt % of the total carbon in the feed.

From a carbon balance perspective, about 75.7 g of the original 87.7 g of carbon is recovered in the final products (biographite, gasoline, diesel, and SAF). The remaining ~12 g of carbon is lost as gaseous or aqueous by‐products from each processing step where we did not track carbon in loss streams. Total losses across the five‐step process sum to 18.73 g (or wt %) and 11.96 g carbon (13.6 wt % carbon). Losses occurred predominantly during coking (2.20 g), where non‐condensable gases are driven off, and hydrotreating (12.99 g), where gaseous and aqueous fractions (listed in Table 3) are collected but did not contribute to the production of the hydrocarbon fuels. Overall, the process achieves a favorable mass and carbon yield, demonstrating the potential of a biomass‐derived feedstock to produce two strategic products (battery‐grade biographite and SAF) from biorefinery residue, thereby offering a more sustainable alternative to traditional petroleum‐based routes.

## Conclusions

Integrated cascading catalytic pyrolysis (ICCP) of glycerol for the production of benzene, toluene, and xylene (BTX) generates a heavy residue that is rich in polycyclic aromatic hydrocarbons (PAHs). This study presents a novel valorization pathway for this PAH‐rich bio‐oil, demonstrating its potential as a drop‐in feedstock for the production of battery‐grade graphite and sustainable aviation fuel (SAF). Through a three‐step thermal treatment of coking at 500 °C, calcination at 1000 °C, and graphitization at 2800 °C, the bio‐oil was transformed into highly crystalline biographite. The coking process produced green bio‐coke (20 wt %) and coke oil (78 wt %). The solid green bio‐coke is a suitable precursor to battery‐grade graphite anode material. The liquid coke oil is highly aromatic and can be hydrotreated as a drop‐in feedstock in existing petroleum refineries. A key advancement of this work is the coking step prior to hydrotreatment, which effectively removes higher molecular weight compounds that would otherwise inefficiently consume hydrogen and form products not suitable for SAF. Hydrotreatment of the coke oil yielded 70 wt % SAF, surpassing previous reports (~50 %)[^63^, ^80^]. The SAF fraction had a high cycloalkane content, boosting energy density compared to paraffinic SAF while still preserving elastomer swelling—a crucial but often overlooked requirement traditionally met by aromatics in drop‐in aviation fuel. The biographite exhibited excellent performance in half‐cell configurations with a reversible specific capacity of 332 mAh g^−1^ active material, the same result demonstrated by a needle coke graphite reference and only slightly lower than a commercial synthetic graphite reference (344 mAh g^−1^). Additionally, the biographite demonstrated high initial coulombic efficiency (>90 %) and excellent rate capability performance in full‐cell configurations, highlighting its potential as a viable alternative to petroleum‐derived synthetic graphite. This study establishes a new pathway in bio‐crude refining of PAH‐rich residues. By co‐producing biographite and SAF, this process presents a compelling alternative to conventional bio‐crude valorization, offering a novel approach to biorefinery optimization.

## Conflict of Interests

Tijmen Vries and Ton Vries have a financial interest in BioBTX. Michael Regula and Zachary A. Combs have a financial interest in Birla Carbon U.S.A., Inc.

1

## Data Availability

The data that support the findings of this study are available from the corresponding author upon reasonable request.
